# Exploring the landscape of adult autism research in psychology: a bibliometric and network analysis

**DOI:** 10.3389/fpsyg.2024.1427090

**Published:** 2024-09-12

**Authors:** Fabio Batista Mota, Luiza Amara Maciel Braga, Bernardo Pereira Cabral

**Affiliations:** ^1^Laboratory of Cellular Communication, Oswaldo Cruz Institute, Oswaldo Cruz Foundation, Rio de Janeiro, Brazil; ^2^Department of Economics, Federal University of Bahia, Salvador, Brazil

**Keywords:** autism spectrum disorder, adults, scientific publications, bibliometrics, network analysis, PRISMA

## Abstract

The global prevalence of autism spectrum disorder (ASD) is increasing. ASD manifests with persistent social communication and interaction challenges, limited interests, and repetitive behaviors. As the scientific literature on ASD in adults varies greatly, mapping the recent global research becomes valuable for enhancing comprehension of this subject. This study aims to map recent global scientific publications on ASD in adults. Using the Preferred Reporting Items for Systematic Reviews and Meta-Analyses, bibliometrics, and network analyses, we assessed 850 articles indexed in the Web of Science Core Collection between 2013 and 2022 assigned to the research area of psychology. Findings indicate an annual average growth of 11.69%. Key keywords include Emotion, Anxiety, and Depression, with Anxiety, Depression, and Mental Health as central nodes in the network. Rehabilitation, Behavioral Sciences, and Psychiatry frequently co-occur, and Psychology, Psychiatry, and ‘Neurosciences and Neurology’ are central nodes in the network of research areas. The United States of America and the United Kingdom lead in publications, with the United Kingdom being the most central country in the network. King’s College London and the University of California are the main research organizations, with King’s College London as the central node in the network. The American Psychiatric Association’s DSM-5-TR was the most cited reference in the period. This comprehensive analysis contributes to understanding the landscape of ASD research in adults, providing insights for future research and fostering collaborations.

## 1 Introduction

The autism spectrum disorder (ASD) involves persistent deficits in social communication and social interaction, as well as restricted areas of interest and repetitive patterns of behavior. Individuals with ASD manifest such symptoms early in life, which may cause significant impairments in daily functioning. This lifelong condition persists into adulthood and can be diagnosed across the lifespan, with symptoms presenting differently in adults and children. The severity of ASD is classified in a range from level 1 to 3, where level 1 means that the individual requires support, and level 3 requires substantial support (American Psychiatric Association, 2013).

According to the World Health Organization (WHO), ASD affects around 1 in 100 children worldwide (WHO, 2023). Much of the research on ASD still focuses on children (Howlin and Magiati, 2017). However, there has been a growing understanding of the importance of studying and addressing the specificities of ASD in adults (Howlin and Magiati, 2017; Howlin, 2021; Brede et al., 2022), considering, e.g., the elevated psychological and social costs (Howlin and Magiati, 2017), the lower quality of the support services compared to those available for children, and the limited outlook for job opportunities and self-sufficient living (Howlin, 2021). Furthermore, individuals with ASD are more likely to develop co-occurring mental (Brede et al., 2022; Forde et al., 2022) and physical health conditions. Nevertheless, knowledge of these aspects and the overall health status of the ASD adult population are still poor (Forde et al., 2022) despite their need for specialized healthcare services (Gilmore et al., 2022a; Malik-Soni et al., 2022), including mental healthcare (Brede et al., 2022; David et al., 2022; Gilmore et al., 2022b). Some common mental conditions associated with ASD are anxiety (Menezes et al., 2022; Galvin and Richards, 2023) and obsessive-compulsive disorder (OCD) (Russell et al., 2016; Forde et al., 2022), while some frequent physical conditions are epilepsy (El Achkar and Spence, 2015; Forde et al., 2022) and sleep disorders (Lugo et al., 2020; Morgan et al., 2020; Forde et al., 2022).

Overall, the current knowledge of adult ASD diagnosis is still limited (Krijnen et al., 2023), as ASD is commonly diagnosed during infancy and early childhood (Lupindo et al., 2022). An early ASD diagnosis plays a critical role in achieving better health outcomes. However, many individuals might not receive such a diagnosis until adulthood (Lupindo et al., 2022; Ghanouni and Seaker, 2023). Although adults face many barriers to efficient diagnosis (Huang et al., 2020), as awareness of this subject develops, more adults are expected to undergo ASD assessment and diagnosis (Huang et al., 2020; Gellini and Marczak, 2023). As late diagnosis postpones access to healthcare (Ghanouni and Seaker, 2023), it comes with great financial and emotional costs to the individual and their families (Lupindo et al., 2022). The factors behind late diagnosis vary. It may include, e.g., compensatory strategies and the masking of autistic traits (Lupindo et al., 2022; Gellini and Marczak, 2023; Krijnen et al., 2023), subtle symptoms (Lupindo et al., 2022; Krijnen et al., 2023), and misinterpretation of autistic traits as other psychiatric disorders (Lupindo et al., 2022).

Different life stages present specific challenges, requiring targeted research to develop effective interventions. As ASD individuals progress into adulthood, their needs and challenges substantially differ from those observed in childhood (Howlin, 2021) as new variables may come into play, such as entry into the workforce (Wehman et al., 2016; Hedley et al., 2017), and marriage (Qutub, 2023). Adults with ASD may face difficulties in, e.g., employment (Schwartzman and Corbett, 2022), and independent living (Howlin, 2021), which are not the focus of interventions designed for children. Therefore, it is important to increase research specifically focused on adults with ASD to both enhance the overall understanding of its particularities and to develop interventions designed to address the challenges they may face across their lifespan.

Over the last 10 years, how has the global research on ASD in adults evolved, and what are the main trends, themes, research areas, and institutional collaborative networks? As know, the scientific literature on ASD in adults varies greatly (Howlin and Magiati, 2017). It ranges from, e.g., non-pharmacological interventions (Speyer et al., 2022), to physical activity and sedentary behavior (Thompson et al., 2023), depression and employment (Schwartzman and Corbett, 2022), and suicidal thoughts (Jachyra et al., 2022). Thus, taking stock of the recent research carried out worldwide would benefit the overall understanding of this subject, providing insights that may inform future research. Thus, this study aims to map the recent scientific publications on ASD in adults. To do so, we carried out a bibliometric and network analysis using metadata of articles related to ASD in adults indexed in the Web of Science Core Collection (WoS), assigned to the psychology field, and published in the last 10 years (2013–2022). Bibliometrics analyzes and measures data and text, especially from bibliographic sources. It involves a statistical examination of, e.g., frequencies, relationships, and trends present in a group of publications covering a given subject. In turn, network analysis assesses co-occurrences among entities in a system represented as a network of interconnected nodes and edges, where the former can represent individuals, organizations, countries, etc., and the latter relationships between them. Bibliometrics and network analysis are commonly employed together in academic research to analyze a variety of aspects related to a scientific subject or field (Comarú et al., 2021; Maciel Braga and Mota, 2021).

As far as we know, our study is the first to map the global publication related to ASD in adults through bibliometrics and network analysis. It contributes to the existing literature, offering an overall landscape of the research produced in this field over the last 10 years. We expect our study to be of interest to ASD-related researchers investigating this disorder in the adult population. By identifying some of the most relevant players producing knowledge in this field and their networks of research collaboration, as well as the main analyzed subjects and research areas and their relationships, our findings may provide insights for future research and foster interinstitutional collaboration related to ASD in adults.

## 2 Methods

We used bibliometrics and network analysis to analyze the metadata of research articles on ASD in adults indexed in WoS. The articles’ records were identified using the following search strategy (or query) applied in the WoS advanced search mode:

TI = ((“Autism*” or “Autistic*” or “Asperger*” or “ASD”) near/3 (“Adult*”)) not (TI = (“Newborn*” or “Neonate*” or “Infant*” or “Child*” or “Adolescen*” or “Teen*” or “Youth*”) or TI = (“*Broad* Autis* Phenotype*” or “*Broad* Autis* Spectrum*” or “autis* trait*” or “Autis* Spectrum* Trait*” or “Autis* Phenotype* trait*”)) and Article (Document Types) and Psychology (Research Areas).

Timespan: 2013-01-01 to 2022-12-31.

The query uses the field tag TI (Title: search for records in the documents’ titles) and includes descriptors related to ASD, adults, infants, children, and adolescents collected in the Medical Subject Headings (MeSH: https://www.ncbi.nlm.nih.gov/mesh/). For simplicity, from now on, these last three descriptors, as well as others related to them, will be called children-related descriptors. The query has two parts. The first searches for records with ASD-related descriptors near adult-related descriptors, with up to three words between them. The second searches for records with children-related descriptors or records with terms related to broad autism phenotype (BAP) or autistic traits. The Boolean operator “not” between the first and second parts of the query excludes from the results (i) the records of articles that include children-related descriptors along with adults and (ii) articles related to adults with no ASD diagnosis. Both BAP and ASD involve certain autistic traits, but the distinction lies in the severity and impact of these traits. ASD represents a clinical diagnosis, while BAP refers to milder or subclinical traits observed in individuals without a formal ASD diagnosis (Bang et al., 2022; Oliveros et al., 2023).

**Figure 1:**
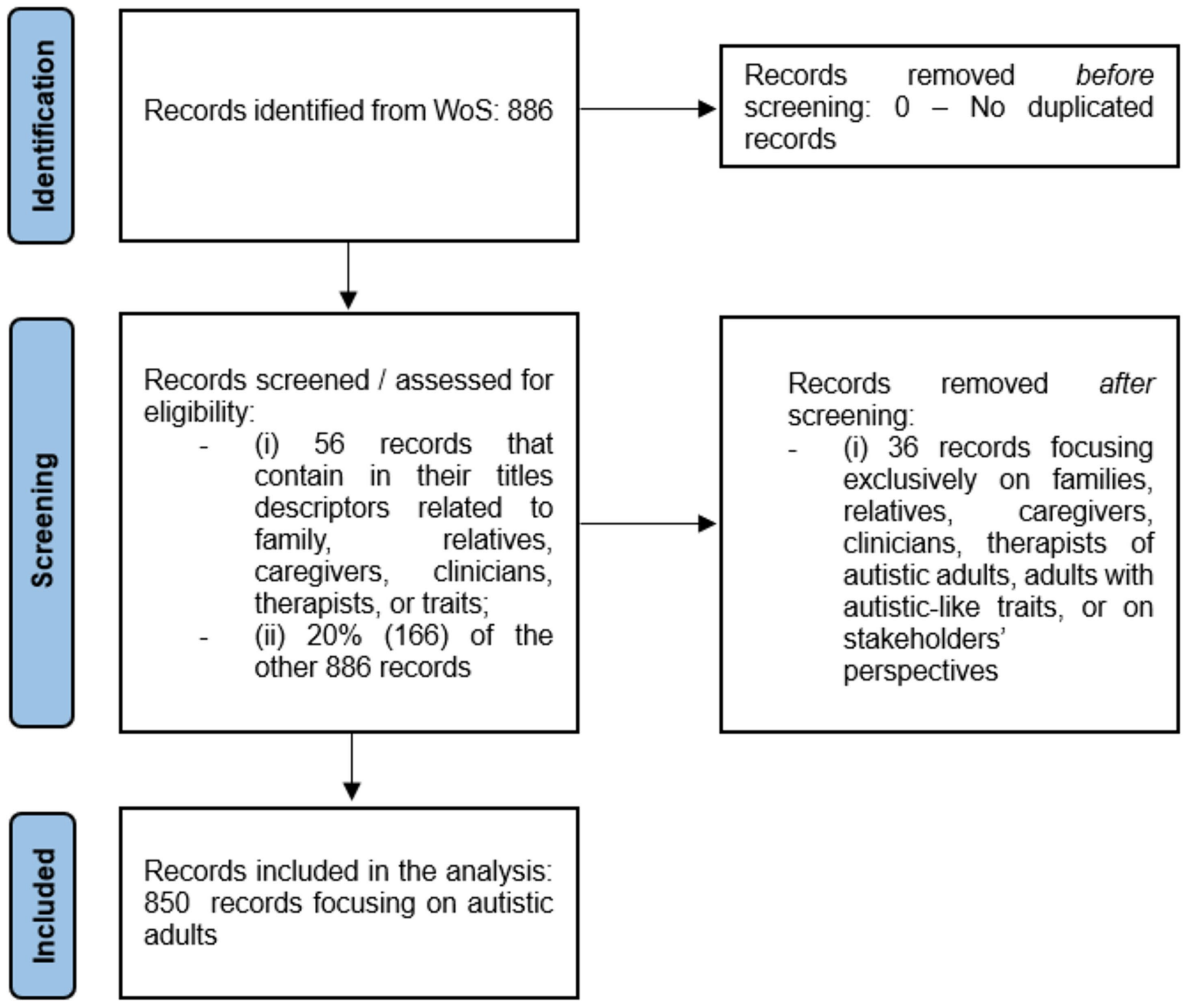
Identification and selection of articles.

The query includes only research articles, and the timespan covers the last ten years (2013–2022) and searches for records assigned to the Research Area (RA) of Psychology. RAs are a subject classification scheme elaborated by WoS, where the articles indexed in its database are assigned to one or more RAs (WoS: https://images.webofknowledge.com/images/help/WOS/hp_research_areas_easca.html). Both the search and data collection were performed on August 30, 2023. We collected 886 records, which were imported to the data/text mining software VantagePoint 11.0 for treatment and analysis. We checked the title, Digital Object Identifier (DOI), and ISI Unique Article Identifier of the articles and found no duplicated records. We included the articles focusing on autistic adults and excluded those without this emphasis. To do so, we searched for records containing descriptors related to family, relatives, caregivers, clinicians, therapists, autistic-like traits, or stakeholders in their titles, and excluded 36 as they do not focus on autistic adults. Then, we screened a 20% random sample of the other records and found no records out of the scope of the study. Thus, the final dataset contains 850 records of articles related to ASD adults. Figure 1 summarizes the identification, screening, and inclusion process of articles, that followed the Preferred Reporting Items for Systematic Reviews and Meta-Analyses (PRISMA) (Page et al., 2021).

From the articles’ metadata collected in WoS, we use publication years, journals, author keywords, research areas, author affiliations, countries, and cited references with a Digital Object Identifier (DOI). Using VantagePoint, we (i) cleaned and standardized authors’ affiliations (or organizations) and authors’ keywords data using the list cleanup tool, general matching rule, and manual cleaning, and (ii) generated co-occurrence matrices of keywords, research areas, countries, and organizations. These matrices were then imported into the software Gephi 0.10 where we generated the networks and calculated the network metrics of degree centrality (DC), weighted degree centrality (WDC), closeness centrality (CC), betweenness centrality (BC), and eigenvector centrality (EC). DC measures the number of direct connections a node has in a network. Nodes with a higher DC are more central regarding direct connections within the network. WDC is similar to DC, but it considers the weight of the connections between nodes (the number of links between two nodes) to assess the importance of a node within the network. CC measures how quickly a node can reach all other nodes in the network. Nodes with a higher CC are more central in terms of proximity to other nodes, indicating a shorter average distance from a given node to all other nodes in the network. BC measures how often a node lies on the shortest paths between other nodes in the network. Nodes with a higher BC may have a greater influence over the flow of information between other nodes in the network. Finally, EC assesses how well-connected a node is with central nodes in the network (Comarú et al., 2021; Maciel Braga and Mota, 2021; Mota et al., 2022). The values of these centrality metrics are available in the Supplementary material. The Fruchterman-Reingold algorithm provided the layout of the networks (Gephi: github.com/gephi/gephi/wiki/Fruchterman-Reingold). Node sizes and colors are given by the WDC and the edges’ thickness by the co-occurrence between nodes. We used GraphPad Prism 8 to elaborate the frequency graphs and combine them with Gephi’s networks. The main journals’ 2022 Impact Factor (IF) was collected in Clarivate’s Journal Citation Reports 2023.^1^

## 3 Results

**Figure 2:**
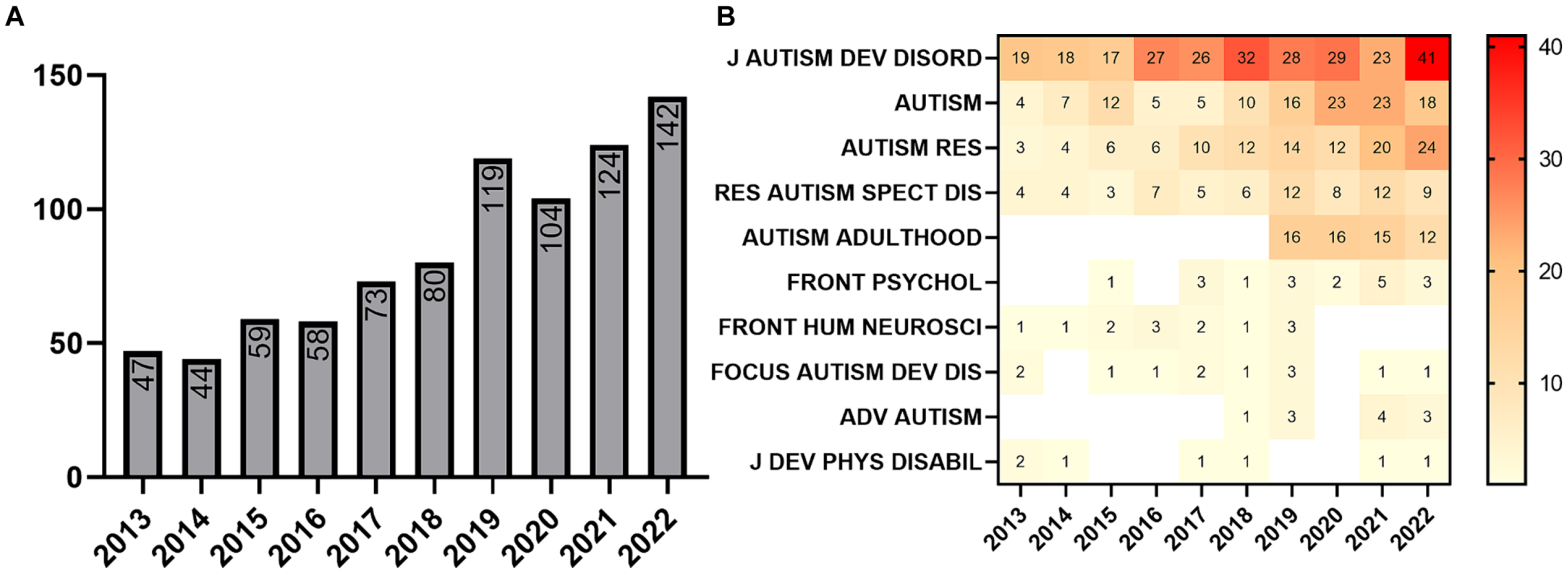
Publication over time and main journals. **(A)** Evolution of annual publishing. **(B)** Evolution of annual publishing by top 10 journals.

Analyzing the number of articles related to ASD in adults over time, we see an increase in publications with an annual average growth of 11.69% and a peak in 2022 (142 articles). In 2019, it surpassed 100 articles for the first time (119 articles). From 2013 to 2022, the increase in publications was 202.13%. Of its 850 records, 57.53% occurred between 2019 and 2022 (Figure 2A). Among the top 10 journals publishing on this topic, the Journal of Autism and Developmental Disorders, from Springer, stands out with 30.59% of publications (260 articles, of which 15.77% (41) were published in 2022), followed by Autism (123; 14.47%; Sage Journals), and Autism Research (111; 13.06%; Wiley) (Figure 2B). One hundred and seven journals published on ASD in adults, with the top 10 concentrating 80.47% of all articles. The 2022 Impact Factor of the top 10 journals ranges from 1.2 (Advances in Autism) to 6.8 (Autism in Adulthood), with a median of 3.35.

**Figure 3:**
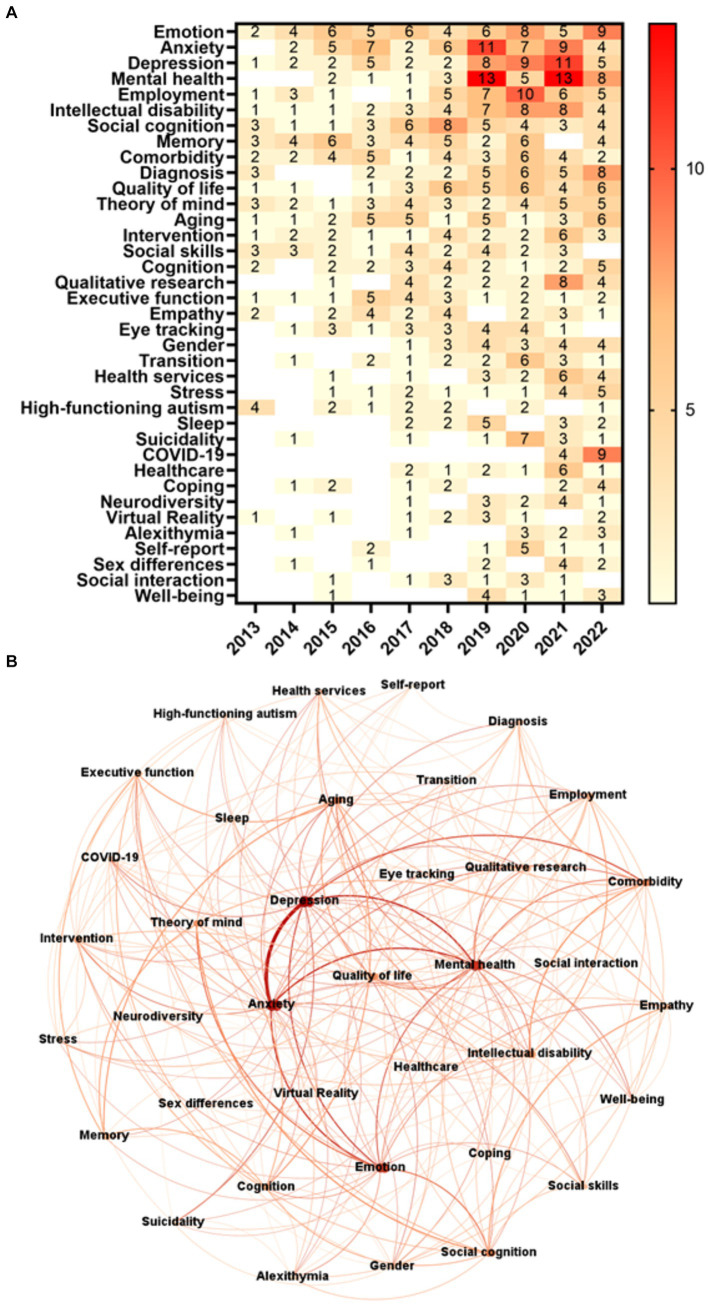
Authors’ keywords. **(A)** Frequency of author’s keywords over time. **(B)** Network of authors’ keywords. Undirected network comprising 37 nodes and 241 edges. **(A,B)** Keywords with a frequency greater than or equal to 10 excluding the ones related to the search strategy applied in WoS (ASD (733), Adults (234), Asperger’s syndrome (51), Autistic adults (35), Adulthood (26), Young adults (24)) and keywords with incomplete meaning [Qualitative (12), and Outcome (10)].

With 55 occurrences, Emotion was the most frequent authors’ keyword of the period 2013–2022, followed by Anxiety (53), Depression (47), and Mental Health (46). Over 50% of these four most frequent keywords occurred in the last 4 years of the series, with Mental Health and Depression standing out with 84.78 and 70.21%, respectively (Figure 3A). The network of authors’ keywords (Figure 3B) shows that, according to WDC, the three most central nodes are Depression (140.0), Anxiety (132.0), and Mental Health (118.0). However, three other centrality metrics put Anxiety as the most central node of the network (EC: 1.0; CC: 0.765957; and DC: 25), with Depression (EC: 0.956458; CC: 0.734694; and DC: 23) ranking second and Mental Health third (EC: 0.891994; CC: 0.720000; and DC: 22). Emotion ranks first in BC (0.073796), with Anxiety (0.073710) and Mental Health (0.060939) respectively in second and third. The most co-cited keywords were Anxiety and Depression, co-occurring 21 times.

**Figure 4:**
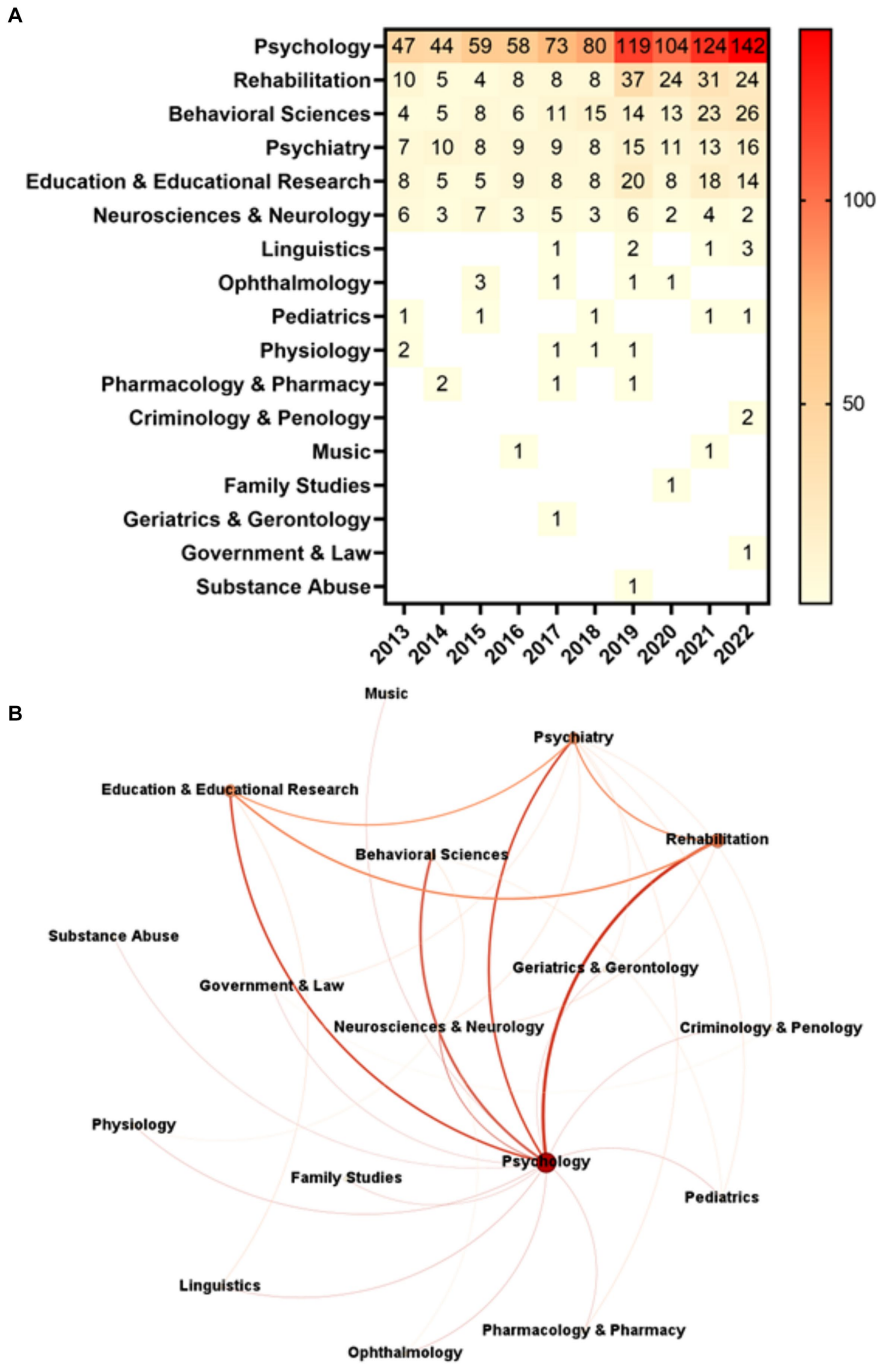
Research Areas. **(A)** Frequency of Research Areas over time (all Research Areas). **(B)** Network of Research Areas (all Research Areas). Undirected ego network comprising 17 nodes and 49 edges. The “ego” represents the RA Psychology and the ego network consists of RAs connected to Psychology.

Psychology is the most frequent RA, as we only retrieved articles’ records that WoS assigned to this RA (Figure 4A). Therefore, its distribution over time is the same as in Figure 1. Rehabilitation (159 records), Behavioral Sciences (125), and Psychiatry (106) are the three most frequent RAs occurring together with Psychology. From 2013 to 2022, the annual average growth rates for these RAs were 9.15, 20.58, and 8.62%, respectively. For the same reason, Psychology is the most central node of the network in any of the five analyzed centrality metrics (DC: 16; WDC: 1,138.0; CC: 1.0; BC: 0.7125; and EC: 1.0). Considering all centrality metrics except WDC (514.0), Psychiatry, the third most RA cited together with Psychology, is the second most central node of the network (DC: 9; CC: 0.695652; BC: 0.101389; and EC: 0.744785). Likewise, except for WDC (120.0), the RA Neurosciences and Neurology – the fifth most cited with Psychology – ranks third in relevance in the network (DC: 6; CC: 0.615385; BC: 0.036111; and EC: 0.552505). WDC puts the RA Rehabilitation as the second most central node (648.0), followed by Education and Educational Research (544.0) (Figure 4B).

**Figure 5:**
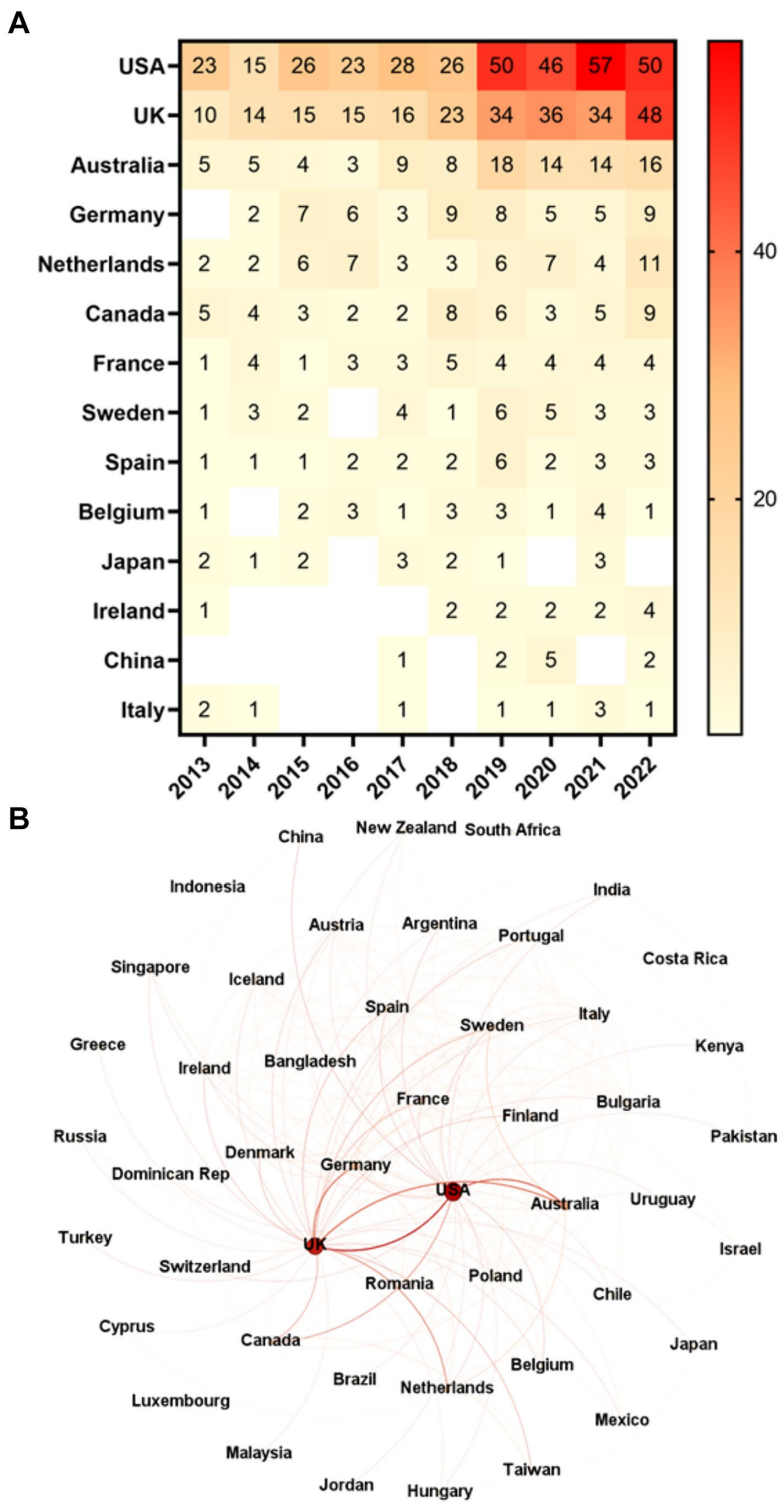
Countries. **(A)** Frequency of countries’ publications over time (countries with a frequency greater than or equal to 10). **(B)** Network of countries (all countries) comprising 48 nodes and 204 edges.

The United States of America (USA) was the most frequent country publishing research results related to adults with ASD (Figure 5A). From 2013 to 2022, its production amounted to 344 articles, representing 40.47% of total publications, and surpassing the United Kingdom (UK; 245; 28.82%), the second country in the rank, by almost 100 articles. In the analyzed period, Australia ranked third with 11.29% (96) of all publications. The network analysis (Figure 5B) shows the UK as the most central node in the network of countries according to all centrality metrics, but WDC, which ranks second with 780.0 (DC: 35; CC: 1.0; BC: 0.811321; and EC: 0.32836). The USA leads in WDC (864.0), ranking second in DC (29), BC (0.728814), and EC (0.247209), and sixteenth in CC (0.691994). Australia only figures among the three most central nodes in WDC (312.0). The most frequent collaborations were between the USA and the UK (20 articles published together), the USA and Australia (18), the UK and Australia (18), and the UK and Germany (18). In 2022, however, the USA and the UK did not publish research results in collaboration. Canada was the country that the USA published the most (4 articles in collaboration), while the most frequent UK partners were the Netherlands (6), Australia (4), and Germany (4).

**Figure 6:**
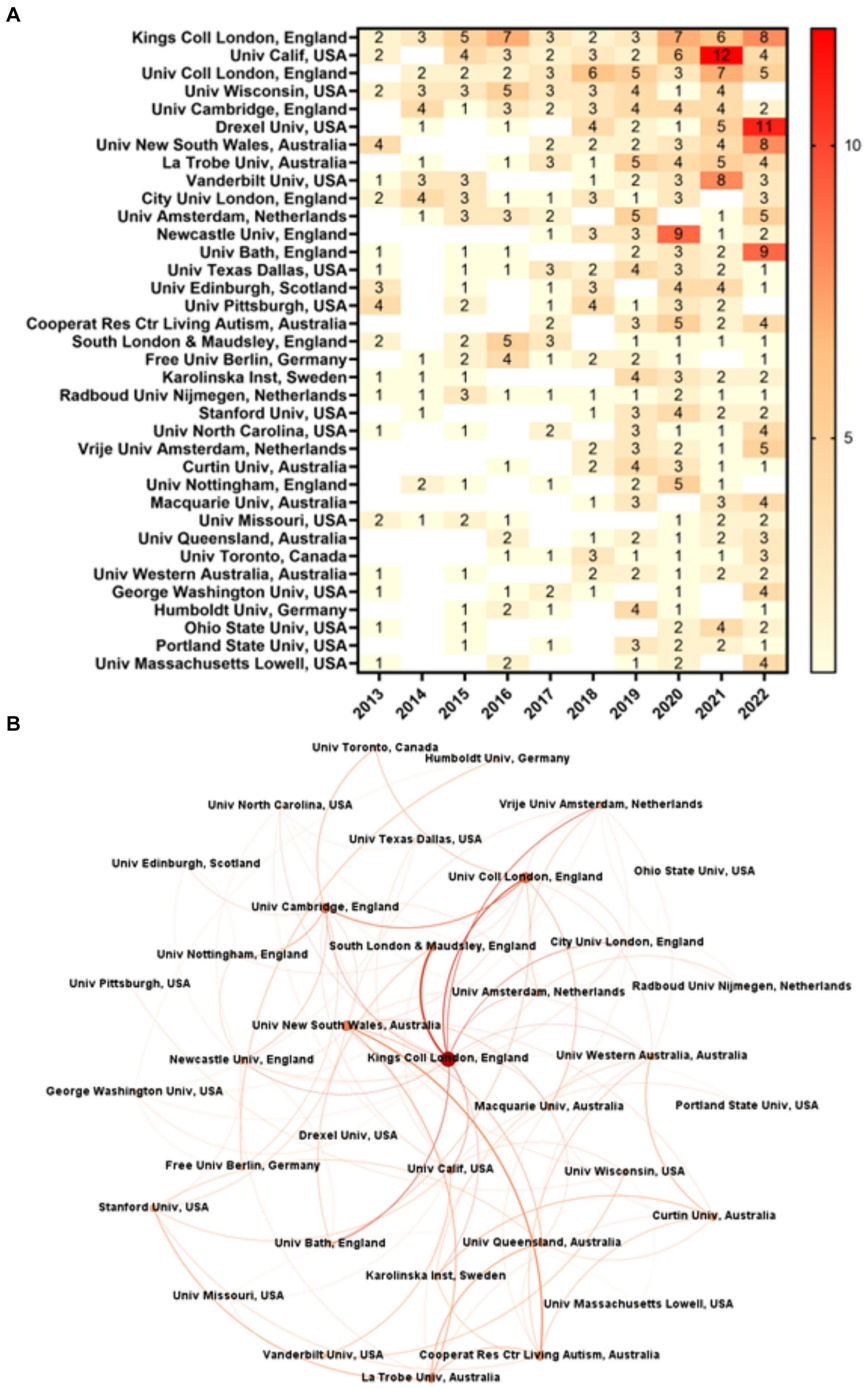
Organizations. **(A)** Frequency of organizations’ publications over time (organizations with a frequency greater than or equal to 10). **(B)** Network of organizations (organizations with a frequency greater than or equal to 10) comprising 36 nodes and 139 edges.

Of the 36 research organizations that have published 10 or more articles between 2013 and 2022, 36.11% are from the USA, 25.00% are based in the UK, and 19.44% in Australia (Figure 6A). Although the USA is the most frequent country publishing on adults with ASD (Figure 5A), King’s College London (KCL) and University College London, both from England, rank first and third among the research organizations, respectively (Figure 6A). The former accounts for 5.41% (46) of total publications and the latter for 4.12%. Ranking second, the University of California (UC) is the most published American organization (4.47%), followed by the University of Wisconsin-Madison (3.29%; fourth). The leading Australian research organizations are the University of New South Wales and La Trobe University, ranking seventh (2.94%) and eighth (2.82%), respectively. The network analysis (Figure 6B) shows KCL as the overall most central node of the network comprising the research organizations with 10 or more articles, ranking first in all centrality metrics but BC, where it is placed in third with 0.146666 (DC: 14; WDC: 110.0; EC: 1.0; CC: 0.586207). Sixth among the publishing organizations, the University of New South Wales from Australia ranks second in DC (12) and EC (0.9797), and third in WDC (62.0). The University College London is among the three most central nodes in WDC (66.0) and EC (0.779845). The UC ranks first in BC (0.223461) and third in CC (0.557377). In turn, the University of Wisconsin-Madison did not appear among the most central nodes. The most frequent research collaborations were between KCL and South London and Maudsley NHS Foundation Trust (SLAM) from England (16 articles published together), KCL and University College London (8), and University College London and the University of Cambridge from England (7).

Cited by 38.71% of all articles in the dataset, the Diagnostic and Statistical Manual of Mental Disorders (DSM-5-TR) (American Psychiatric Association, 2013) from the American Psychiatric Association (APA) is the most cited reference, followed by Baron-Cohen et al. (2001) (27.29%), and Lord et al. (2000) (15.06%) (Table 1). About 46% of the 13 top 10 cited references are from the Journal of Autism and Developmental Disorders. Considering the Web of Science, Lord et al. (1994) is the most cited (6,652). Lord et al. (2000) and Baron-Cohen et al. (2001) rank second (5,523) and third (4,030) respectively. All three most cited references in Web of Science were published by the Journal of Autism and Developmental Disorders.

**Table 1:** Top 10 most cited references in the 850 articles and their number of citations in WoS.

Times cited (Dataset)	Times cited (WoS)	Pub. year	Authors	Title	Source
329	–	2013	American Psychiatric Association	Diagnostic and Statistical Manual of Mental Disorders (DSM-5-TR)	American Psychiatric Association
232	4,030	2001	Baron-Cohen, Simon, et al.	The Autism-Spectrum Quotient (AQ): Evidence from Asperger syndrome/high-functioning autism, males and females, scientists and mathematicians	J Autism Dev Disord
128	5,523	2000	Lord, Catherine, et al.	The Autism Diagnostic Observation Schedule-Generic: A standard measure of social and communication deficits associated with the spectrum of autism	J Autism Dev Disord
107	6,652	1994	Lord, Catherine, et al.	Autism Diagnostic Interview-Revised: a revised version of a diagnostic interview for caregivers of individuals with possible pervasive developmental disorders	J Autism Dev Disord
102	579	2015	Croen, Lisa, et al.	The health status of adults on the autism spectrum	Autism
96	1,054	2004	Howlin, Patricia, et al.	Adult outcome for children with autism	J Child Psychol Psyc
76	539	2009	Hofvander, Björn, et al.	Psychiatric and psychosocial problems in adults with normal-intelligence autism spectrum disorders	Bmc Psychiatry
68	573	2012	Shattuck, Paul, et al.	Postsecondary Education and Employment Among Youth with an Autism Spectrum Disorder	Pediatrics
54	276	2016	Lever, Anne and Geurts, Hilde	Psychiatric Co-occurring Symptoms and Disorders in Young, Middle-Aged, and Older Adults with Autism Spectrum Disorder	J Autism Dev Disord
54	527	2005	Woodbury-Smith, Marc, et al.	Screening adults for Asperger syndrome using the AQ: A preliminary study of its diagnostic validity in clinical practice	J Autism Dev Disord
54	–	2008	Braun, Virginia, and Clarke Victoria	Using thematic analysis in psychology	Qual Res Psychol
52	263	2013	Joshi, Gagan, et al.	Psychiatric Comorbidity and Functioning in a Clinically Referred Population of Adults with Autism Spectrum Disorders: A Comparative Study	J Autism Dev Disord
52	475	2016	Hirvikoski, Tatja, et al.	Premature mortality in autism spectrum disorder	BJPsych

Only cited references with DOI (19,397; 79.12% of total). No information in the column “Times cited (WoS)” means the document is not indexed in WoS.

## 4 Discussion

As seen, the overall most central keywords in the authors’ network were Anxiety and Depression, which were co-cited 21 times. Across the lifespan, individuals with ASD face elevated risks of anxiety disorders, marked by disproportionate responses to potential threats (Menezes et al., 2022), which can affect their mental health (Ghanouni and Quirke, 2023). ASD adults are at a higher risk of developing anxiety disorders due to aspects such as deficits in social communication and efforts to participate in specific social situations by camouflaging autistic traits (Menezes et al., 2022). Examples of recent publications on ASD in adults where the keywords anxiety and depression were cited together include an investigation of mental health experiences and support received (Crane et al., 2019), an assessment of the prevalence of depression, anxiety, and suicidality, and the connections between social difficulties and mental health (Dow et al., 2021), and the links between loneliness, friendship, and emotional functioning (Mazurek, 2014).

The Coronavirus disease 2019 (COVID-19) pandemic caused a burst of issues related to mental health, including anxiety and depression (Fancourt et al., 2021), among many other consequences for the health of the global population. Concerning the ASD adult population, the consequences of the pandemic were significant (Maljaars et al., 2023; Scheeren et al., 2023). They were expressed in higher rates of infection and hospitalization, interruption of healthcare and support services, and social restrictions (Scheeren et al., 2023), with a predominantly negative impact on mental health (Maljaars et al., 2023; Scheeren et al., 2023) as a consequence of an increase in stress, anxiety, and depressive symptoms (Maljaars et al., 2023). In that sense, we expected a stronger correlation between two of the most frequent authors’ keywords (Anxiety and Depression) and COVID-19 in the publications of the period 2020–2022. However, the keyword COVID-19 occurred for the first time in 2021 (4 records) and amounts to just 13 records. Additionally, the keywords Anxiety, Depression, and COVID-19 co-occurred in only one article about the effects of the initial stages of COVID-19 on the mental health of ASD adults in the UK (Bundy et al., 2022). Therefore, exploring this subject further from our collected data was not possible.

In the network of RAs, besides Psychology, the relevance of Psychiatry, and “Neurosciences and Neurology” was expected since historically ASD research has been commonly located in these scientific fields (Kourti, 2021). However, these three RAs co-occurred together in only two articles (Crivelli and Rocca, 2013; Avirame et al., 2017). Analyzing by pairs of RAs, Rehabilitation ranks second in the number of co-occurrences, with Psychiatry ranking third. Recent examples of research focusing on ASD adults and assigned at the same time to Psychology and Rehabilitation are the investigation of living arrangements and satisfaction levels with current living situations (Song et al., 2022), the identification of health and social service priorities, and the factors influencing the receipt of these services (Jose et al., 2021), and the evaluation of the effects of teaching ASD adults with intellectual disability in leisure activities using mobile devices (Nepo et al., 2021). For its part, examples of studies on adults with ASD assigned at the same time to Psychology and Psychiatry are the prevalence of co-occurring psychiatric and neurological diagnosis (Underwood et al., 2023), the interplay between access to professional and social support, discrimination, and victimization in explaining psychological distress (Jeanneret et al., 2022), and the character strengths profiles as a potential means to identify interventions focused on strengths to improve well-being outcomes (Nocon et al., 2022).

Just as the growing number of annual scientific publications on ASD, the global prevalence of ASD appears to be on the rise, although its rates vary significantly among studies (World Health Organization, 2013; Salari et al., 2022; Zeidan et al., 2022). Epidemiological data reviewed by the WHO in a report published in 2013 indicated that the median global prevalence of ASD was 1 in 160 children, contributing to over 7.6 million disability-adjusted life years and comprising 0.3% of the global burden of disease (World Health Organization, 2013). Among other things, a more recent increase in awareness and changes in diagnostic criteria may have impacted the global rates of ASD diagnosis (Solmi et al., 2022). Some recent studies have reported, e.g., that the prevalence of ASD is 0.6%(Salari et al., 2022) and 0.72%(Talantseva et al., 2023) of the global population. It should be noted, however, that data from many low- and middle-income countries is usually missing, as much of the data on ASD prevalence comes from research carried out in high-income countries (World Health Organization, 2013).

In the USA, the Centers for Disease Control and Prevention (CDC) periodically releases estimates of ASD prevalence in children based on surveillance data reviewed by the Autism and Developmental Disabilities Monitoring (ADDM) Network. As of the latest available data, the estimated prevalence of ASD in 8-year-old children in the USA was 1 in 36 and was about four times more likely in boys than in girls (ADDM Network, 2023). More recently, there has been increasing attention on diagnosing ASD in females, a group that may have been overlooked in previous ASD epidemiological and clinical research (Solmi et al., 2022). In adults, the prevalence of ASD would be 2.21% according to the CDC (2022). In England, recent studies indicate that the prevalence of ASD in the overall population may range from, e.g., 0.82%(O’Nions et al., 2023) to 1.76%(Roman-Urrestarazu et al., 2021).

The prevalence of ASD diagnosis in early childhood (18 to 24 months) has been increasing over the years (Okoye et al., 2023), which in part explains differences in prevalence rates between children and adults. Some other contributors to differences in prevalence are underdiagnosis in adults (O’Nions et al., 2023), and unique challenges in assessing ASD in adulthood, such as the availability of the individual’s developmental history and social barriers (Gellini and Marczak, 2023). Limitations of current diagnostic tools are also a challenge. For example, the Autism Diagnostic Observation Schedule, second edition (ADOS-2) may not detect more subtle symptoms of ASD as these individuals usually develop coping mechanisms that may mask their deficits in social communication and social interaction (Curnow et al., 2023). In higher-functioning ASD adults, some examples of coping mechanisms are militancy, intellectualization, and humor (Dachez and Ndobo, 2018). Despite these challenges, over the past decades the overall increased awareness of ASD and the broadening of diagnostic criteria have led to a period where many adult individuals not considered for ASD during childhood are now seeking diagnosis (Gellini and Marczak, 2023). This is, however, a transitioning period as early diagnosis in childhood is expected to become more frequent over time (Okoye et al., 2023). As a consequence, adults seeking ASD diagnosis will probably be less common in the near future.

Despite the great prominence of the USA in the global publication on ASD in adults, the network analysis showed the UK as the most central node in the network of countries. Unexpectedly, the USA led only in WDC, while the UK led in all other metrics. Overall, these findings suggest that the USA is not as collaborative with other countries as the UK in producing knowledge on this subject. Leading in WDC means that the USA is the most influential country in terms of the strength of its connections. For its part, the UK would be the most relevant in terms of direct interactions with other countries (according to DC), connections to other well-connected countries (EC), the average distance to reach other countries (CC), and as an intermediary in connecting other countries (BC). The USA collaborates the most with the UK and vice versa. Collaboratively, researchers from these nations contribute to the body of knowledge on various topics related to ASD in adults. Their research includes, for example, the impact of sensory sensitivity and intolerance of uncertainty on anxiety (Normansell-Mossa et al., 2021), the interplay of lifetime and perceived stress, social support, loneliness, and health (Moseley et al., 2021), the connections between adverse life events, parental mental health, and emotional and behavioral outcomes (Hollocks et al., 2021), and psychometric testing (Nicolaidis et al., 2021a, 2021b).

KCL ranked first in published articles with over 5% of total publications and was the most central node in the network of research organizations. It ranked first in DC, WDC, EC, and CC, meaning that this organization is highly connected, has strong and influential connections, and is positioned close to other organizations in the network. KCL is a public research university located in London, England. Established in 1829, it is one of the most prestigious universities in the UK and a world-leading research organization (KCL: https://www.kcl.ac.uk/about). It has been involved in ASD research for many years and has various initiatives and research centers focusing on this subject. An example is the MRC Centre for Neurodevelopmental Disorders, which investigates the formation, development, and maturation of the brain, examining how disruptions in these processes can lead to neurodevelopmental disorders (MRC: https://devneuro.org/cndd/index.php). Another one is the Institute of Psychiatry, Psychology and Neuroscience (IoPPN). The IoPPN is one of Europe’s leading centers for psychiatric, psychological, and neuroscience research and education (IoPPN: https://www.kcl.ac.uk/ioppn/about). Some research centers or groups related to ASD and associated with the IoPPN are the Autism and Development Research Group, the Global Research on Autism and other Developmental Disabilities (GLAD) Lab, and the ReSpect Lab: Researching Autism across the Spectrum and Lifespan (IoPPN: https://www.kcl.ac.uk/mental-health-and-psychological-sciences/research/groups?keyword=autism).

As seen, the most frequent collaborations in the network of organizations occurred between KCL and SLAM (16 articles). The subjects of their collaboration vary greatly. It ranges from contact with the criminal justice system (Blackmore et al., 2022), to trauma and post-traumatic stress disorder (PTSD) (Rumball et al., 2020), and cognition and behavior (Johnston et al., 2019). The SLAM is the largest mental health trust in the UK, providing a wide range of mental health services to the UK’s population. Among others, it has a research collaboration with the IoPPN (SLAM: https://slam.nhs.uk/who-we-are).

Second in number of publications with over 4% of publications, the UC is a prestigious public university system in California, USA, with multiple campuses across the state. Given the decentralized nature of the UC system, different campuses may house their own research centers, programs, or initiatives focused on ASD research. An example is the Medical Investigation of Neurodevelopmental Disorders (MIND Institute) at UC Davis, known for its research on neurodevelopmental disorders, including ASD (MIND: https://give.ucdavis.edu/MIND). The UC ranked first in BC and third in CC, indicating that it is a key intermediary in connecting other organizations and significantly influences the flow of information in the network. It collaborated the most with Vanderbilt University (5 articles). In 2022, they published two collaborative articles about sleep disturbances and depressive symptoms (Lampinen et al., 2022), and employment changes and depressive symptoms (Taylor et al., 2022).

Finally, the APA’s DSM-5 (American Psychiatric Association, 2013), cited by about 40% of the articles comprising our dataset, is perhaps one of the most influencing academic references on ASD worldwide. Today, APA has over thirty-eight thousand members globally and is a world-leading psychiatric organization engaging in practice, research, and academia across diverse patient populations in over 100 countries (APA: https://www.psychiatry.org/about-apa). The APA’s perceptions of ASD changed over time. For example, from the publication of the DSM-III in 1980 to the DSM-IV in 2000, and to the DSM-5 in 2013, the delay in language development was no longer considered a core symptom in the diagnosis, and the diagnostic category of Asperger’s disorder was removed (Howlin, 2021). Asperger’s syndrome is described as a condition similar to ASD, involving social interaction difficulties and repetitive behaviors but with no typical delay in language or cognitive development, and with most individuals having normal intelligence (World Health Organization, 1992). Ranking second and third, with about 27 and 15% of citations, respectively, we had Baron-Cohen et al. (2001) and Lord et al. (2000). The former reports a new instrument to assess the extent to which an intellectually typical adult exhibits traits associated with ASD, the Autism-Spectrum Quotient (AQ) (Baron-Cohen et al., 2001), and the latter is an application of the Autism Diagnostic Observation Schedule-Generic (ADOS-G), a standardized, semi-structured assessment to evaluate social interaction, communication, play, and imaginative use of materials in individuals under suspicion of ASD (Lord et al., 2000). Today, AQ (Golan et al., 2023; Raul et al., 2023) and ADOS-G (Chang et al., 2023; Rasero et al., 2023) are still in use, with the former getting a second edition in 2012 (ADOS-2) (Chang et al., 2023).

## 5 Conclusion

The increasing prevalence of ASD worldwide (Zeidan et al., 2022; Talantseva et al., 2023) underscores the need to deepen scientific understanding and public awareness of this complex condition (Huang et al., 2020), which significantly contributes to the global burden of mental disorders (Solmi et al., 2022). Employing bibliometric and network analysis techniques, this study assessed 850 records of articles on ASD in adults published between 2013 and 2022. The findings revealed a positive annual average growth in the period, with prominent journals such as the Journal of Autism and Developmental Disorders and Autism leading the dissemination of research results on ASD in adults. Key keywords, including Emotion, Anxiety, and Depression, highlight central themes in the network. Psychology, Psychiatry, and “Neurosciences and Neurology” emerged as central RAs in the network. Over the period, the scientific landscape was led by the USA and the UK, with the latter as the most central country in the network. KCL and UC emerged as world-leading organizations publishing on ASD in adults, with KCL standing out as the central node in the network. The APA’s DSM-5-TR played a central role in the production of knowledge, serving as a framework on which many researchers based their understanding of ASD. We hope our study can enrich the comprehension of prevalent research topics, key institutional contributors, and research collaborative dynamics in this diverse field. The insights derived may catalyze future research initiatives and foster collaborative efforts among institutions.

This study has some limitations. Defining a search strategy implies a trade-off between precision and coverage. In this study, in addition to the specific interest in academic production related to psychology, choosing to analyze only records associated with psychology was a methodological choice to increase precision, providing more consistent results than we would obtain using a broad strategy. Anyway, psychology is by far the most frequent RA assigned to articles on ASD in adults (see search strategy in the Supplementary material). We also excluded from the analysis articles focusing on BAP, family, relatives, caregivers, clinicians, therapists, etc. involved with autismASD in adults. This was needed to increase the reliability of the results as well as to reduce the screening of records unlikely to meet the study’s purpose, inclusion and exclusion criteria. Some may consider the use of only one database as a limitation. It is, however, a common practice in bibliometric and network analysis studies, considering, e.g., the difficulties in merging metadata of different sources, the lack of data standardization in related fields, the reduction of data coverage due to missing fields in some databases, etc. (Moral-Muñoz et al., 2020). Furthermore, the WoS is well known in the bibliometric and network analysis field, being widely used to produce such studies (Birkle et al., 2020). Also, WoS is well acknowledged for its large coverage, and for indexing high-standard journals in all areas of knowledge. In addition, only journals indexed in WoS are assigned with Impact Factor, considered a worldwide standard quality indicator in scientific publishing (Birkle et al., 2020). In some cases, however, the merging of different databases is needed. For example, when the timespan of publishing is too long, some records may predate the coverage period of a given database (Mota et al., 2022).

## Data Availability

The data analyzed in this study is subject to the following licenses/restrictions: The datasets used and/or analysed during the current study are available from the corresponding author on reasonable request. Requests to access these datasets should be directed to fabio.mota@fiocruz.br.

## Footnotes

^1^ http://jcr.clarivate.com/

## References

[ref1] ADDM Network (2023). Key Findings from the ADDM Network. Available at: https://www.cdc.gov/ncbddd/autism/pdf/Key_Findings_508.pdf (Accessed November 26, 2023).

[ref2] American Psychiatric Association (2013). Diagnostic and statistical manual of mental disorders. Washington, DC: American Psychiatric Association.

[ref3] Avirame K. Stehberg J. Todder D. (2017). Enhanced cognition and emotional recognition, and reduced obsessive compulsive symptoms in two adults with high-functioning autism as a result of deep transcranial magnetic stimulation (dTMS): a case report. Neurocase 23, 187–192. doi: 10.1080/13554794.2017.1361451, PMID: 28786315

[ref4] Bang P. Strömberg M. Meera S. S. Igelström K. (2022). Brief report: the broad autism phenotype in Swedish parents of children with and without autism Spectrum conditions. J. Autism Dev. Disord. 52, 4575–4582. doi: 10.1007/s10803-021-05302-3, PMID: 34609695 PMC9508042

[ref5] Baron-Cohen S. Wheelwright S. Skinner R. Martin J. Clubley E. (2001). The autism-Spectrum quotient (AQ): evidence from Asperger syndrome/high-functioning autism, males and females, scientists and mathematicians. J. Autism Dev. Disord. 31, 5–17. doi: 10.1023/A:1005653411471, PMID: 11439754

[ref6] Birkle C. Pendlebury D. A. Schnell J. Adams J. (2020). Web of science as a data source for research on scientific and scholarly activity. Quant. Sci. Stud. 1, 363–376. doi: 10.1162/qss_a_00018

[ref7] Blackmore C. E. Woodhouse E. L. Gillan N. Wilson E. Ashwood K. L. Stoencheva V. . (2022). Adults with autism spectrum disorder and the criminal justice system: an investigation of prevalence of contact with the criminal justice system, risk factors and sex differences in a specialist assessment service. Autism 26, 2098–2107. doi: 10.1177/13623613221081343, PMID: 35261275 PMC9596951

[ref8] Brede J. Cage E. Trott J. Palmer L. Smith A. Serpell L. . (2022). “We have to try to find a way, a clinical bridge”- autistic adults’ experience of accessing and receiving support for mental health difficulties: a systematic review and thematic meta-synthesis. Clin. Psychol. Rev. 93:102131. doi: 10.1016/j.cpr.2022.102131, PMID: 35180632

[ref9] Bundy R. Mandy W. Crane L. Belcher H. Bourne L. Brede J. . (2022). The impact of early stages of COVID-19 on the mental health of autistic adults in the United Kingdom: a longitudinal mixed-methods study. Autism 26, 1765–1782. doi: 10.1177/13623613211065543, PMID: 35083922 PMC9483192

[ref10] CDC (2022). CDC releases first estimates of the number of adults living with autism Spectrum disorder in the United States. CDC Key Findings. Available at: https://www.cdc.gov/ncbddd/autism/features/adults-living-with-autism-spectrum-disorder.html (Accessed December 3, 2023).

[ref11] Chang J. C. Lai M. C. Chien Y. L. Cheng C.-Y. Wu Y.-Y. Gau S. S.-F. (2023). Psychometric properties of the mandarin version of the autism diagnostic observation schedule-generic. J. Formos. Med. Assoc. 122, 574–583. doi: 10.1016/j.jfma.2023.01.008, PMID: 36732136

[ref12] Comarú M. W. Lopes R. M. Braga L. A. M. Batista Mota F. Galvão C. (2021). A bibliometric and descriptive analysis of inclusive education in science education. Stud. Sci. Educ. 57, 241–263. doi: 10.1080/03057267.2021.1897930

[ref13] Crane L. Adams F. Harper G. Welch J. Pellicano E. (2019). ‘Something needs to change’: mental health experiences of young autistic adults in England. Autism 23, 477–493. doi: 10.1177/1362361318757048, PMID: 29415558

[ref14] Crivelli B. Rocca P. (2013). Differential diagnosis between schizophrenia and autism in adulthood: a case report. Neurocase 19, 604–612. doi: 10.1080/13554794.2012.713492, PMID: 22934940

[ref15] Curnow E. Utley I. Rutherford M. Johnston L. Maciver D. (2023). Diagnostic assessment of autism in adults – current considerations in neurodevelopmentally informed professional learning with reference to ADOS-2. Front. Psych. 14:1258204. doi: 10.3389/fpsyt.2023.1258204, PMID: 37867776 PMC10585137

[ref16] Dachez J. Ndobo A. (2018). Coping strategies of adults with high-functioning autism: a qualitative analysis. J. Adult Dev. 25, 86–95. doi: 10.1007/s10804-017-9278-5

[ref17] David N. Dückert S. Gewohn P. König H. Rahlff P. Erik F. . (2022). Mixed-methods investigation of barriers and needs in mental healthcare of adults with autism and recommendations for future care (BarrierfreeASD): study protocol. BMJ Open 12:e061773. doi: 10.1136/bmjopen-2022-061773, PMID: 35998965 PMC9403111

[ref18] Dow D. Morgan L. Hooker J. L. Michaels M. S. Joiner T. E. Woods J. . (2021). Anxiety, depression, and the interpersonal theory of suicide in a community sample of adults with autism Spectrum disorder. Arch. Suicide Res. 25, 297–314. doi: 10.1080/13811118.2019.1678537 31656121

[ref19] El Achkar C. M. Spence S. J. (2015). Clinical characteristics of children and young adults with co-occurring autism spectrum disorder and epilepsy. Epilepsy Behav. 47, 183–190. doi: 10.1016/j.yebeh.2014.12.022, PMID: 25599987

[ref20] Fancourt D. Steptoe A. Bu F. (2021). Trajectories of anxiety and depressive symptoms during enforced isolation due to COVID-19 in England: a longitudinal observational study. Lancet Psychiatry 8, 141–149. doi: 10.1016/S2215-0366(20)30482-X, PMID: 33308420 PMC7820109

[ref21] Forde J. Bonilla P. M. Mannion A. Coyne R. Haverty R. Leader G. (2022). Health status of adults with autism Spectrum disorder. Rev. J. Autism Dev. Disord. 9, 427–437. doi: 10.1007/s40489-021-00267-6

[ref22] Galvin J. Richards G. (2023). Health anxiety in autistic adults. Res. Autism Spectr. Disord. 104:102146. doi: 10.1016/j.rasd.2023.102146

[ref23] Gellini H. Marczak M. (2023). “I always knew I was different”: experiences of receiving a diagnosis of autistic Spectrum disorder in adulthood-a Meta-ethnographic systematic review. Rev. J. Autism Dev. Disord. 1–20. doi: 10.1007/s40489-023-00356-837363697

[ref24] Ghanouni P. Quirke S. (2023). Resilience and coping strategies in adults with autism Spectrum disorder. J. Autism Dev. Disord. 53, 456–467. doi: 10.1007/s10803-022-05436-y, PMID: 35079928 PMC8788904

[ref25] Ghanouni P. Seaker L. (2023). What does receiving autism diagnosis in adulthood look like? Stakeholders’ experiences and inputs. Int. J. Ment. Health Syst. 17:16. doi: 10.1186/s13033-023-00587-6, PMID: 37291614 PMC10251666

[ref26] Gilmore D. Krantz M. Weaver L. Hand B. N. (2022a). Healthcare service use patterns among autistic adults: a systematic review with narrative synthesis. Autism 26, 317–331. doi: 10.1177/13623613211060906, PMID: 34881676

[ref27] Gilmore D. Longo A. Krantz M. Radford D. Hand B. N. (2022b). Five ways providers can improve mental healthcare for autistic adults: a review of mental healthcare use, barriers to care, and evidence-based recommendations. Curr. Psychiatry Rep. 24, 565–571. doi: 10.1007/s11920-022-01362-z, PMID: 35969335 PMC9376572

[ref28] Golan O. Terner M. Israel-Yaacov S. Allison C. Baron-Cohen S. (2023). The autism-Spectrum quotient-Hebrew version: psychometric properties of a full and a short form, adapted for DSM-5. Autism 27, 796–807. doi: 10.1177/13623613221117020, PMID: 36053012 PMC10074759

[ref29] Hedley D. Uljarević M. Cameron L. Halder S. Richdale A. Dissanayake C. (2017). Employment programmes and interventions targeting adults with autism spectrum disorder: a systematic review of the literature. Autism 21, 929–941. doi: 10.1177/1362361316661855, PMID: 27542395

[ref30] Hollocks M. J. Meiser-Stedman R. Kent R. Lukito S. Briskman J. Stringer D. . (2021). The association of adverse life events and parental mental health with emotional and behavioral outcomes in young adults with autism spectrum disorder. Autism Res. 14, 1724–1735. doi: 10.1002/aur.2548, PMID: 34076371

[ref31] Howlin P. (2021). Adults with autism: changes in understanding since DSM-111. J. Autism Dev. Disord. 51, 4291–4308. doi: 10.1007/s10803-020-04847-z, PMID: 33474661 PMC8531125

[ref32] Howlin P. Magiati I. (2017). Autism spectrum disorder: outcomes in adulthood. Curr. Opin. Psychiatry 30, 69–76. doi: 10.1097/YCO.0000000000000308 28067726

[ref33] Huang Y. Arnold S. R. C. Foley K. R. Trollor J. N. (2020). Diagnosis of autism in adulthood: a scoping review. Autism 24, 1311–1327. doi: 10.1177/1362361320903128, PMID: 32106698

[ref34] Jachyra P. Lai M. C. Zaheer J. Fernandes N. Dale M. Sawyer A. . (2022). Suicidal thoughts and Behaviours among autistic adults presenting to the psychiatric emergency department: an exploratory chart review. J. Autism Dev. Disord. 52, 2367–2375. doi: 10.1007/s10803-021-05102-9, PMID: 34128145 PMC9021086

[ref35] Jeanneret N. Courcy I. Caron V. Giroux M. Guerrero L. Ouimet M. . (2022). Discrimination and victimization as mediators between social support and psychological distress in autistic adults. Res. Autism Spectr. Disord. 98:102038. doi: 10.1016/j.rasd.2022.102038

[ref36] Johnston K. Murray K. Spain D. Walker I. Russell A. (2019). Executive function: cognition and behaviour in adults with autism Spectrum disorders (ASD). J. Autism Dev. Disord. 49, 4181–4192. doi: 10.1007/s10803-019-04133-7, PMID: 31281952 PMC6751156

[ref37] Jose C. George-Zwicker P. Bouma A. Tardif L. Pugsley D. Bélanger M. . (2021). The associations between clinical, social, financial factors and unmet needs of autistic adults: results from an observational study. Autism Adulthood 3, 266–274. doi: 10.1089/aut.2020.0027, PMID: 36605370 PMC8992900

[ref38] Kourti M. (2021). A critical realist approach on autism: ontological and epistemological implications for knowledge production in autism research. Front. Psychol. 12:713423. doi: 10.3389/fpsyg.2021.713423, PMID: 35002826 PMC8732992

[ref39] Krijnen L. J. G. Greaves-Lord K. Mandy W. Mataw K. J. S. Hartog P. Begeer S. (2023). How well can we diagnose autism in adults? Evaluating an informant-based interview: the Dutch developmental, dimensional and diagnostic interview – adult version (3Di-adult). J. Autism Dev. Disord. 54, 3492–3503. doi: 10.1007/s10803-023-06069-5, PMID: 37530914 PMC11362255

[ref40] Lampinen L. A. Zheng S. Taylor J. L. Adams R. E. Pezzimenti F. Asarnow L. D. . (2022). Patterns of sleep disturbances and associations with depressive symptoms in autistic young adults. Autism Res. 15, 2126–2137. doi: 10.1002/aur.2812, PMID: 36082844 PMC9677950

[ref41] Lord C. Risi S. Lambrecht L. Cook E. H. Jr. Leventhal B. L. DiLavore P. C. . (2000). The autism diagnostic observation schedule-generic: a standard measure of social and communication deficits associated with the spectrum of autism. J. Autism Dev. Disord. 30, 205–223. doi: 10.1023/A:1005592401947, PMID: 11055457

[ref42] Lord C. Rutter M. Le Couteur A. Free Hospital R. (1994). Autism diagnostic interview-revised: a revised version of a diagnostic interview for caregivers of individuals with possible pervasive developmental disorders. J. Autism Dev. Disord. 24, 659–685. doi: 10.1007/BF02172145 7814313

[ref43] Lugo J. Fadeuilhe C. Gisbert L. Setien I. Delgado M. Corrales M. . (2020). Sleep in adults with autism spectrum disorder and attention deficit/hyperactivity disorder: a systematic review and meta-analysis. Eur. Neuropsychopharmacol. 38, 1–24. doi: 10.1016/j.euroneuro.2020.07.004, PMID: 32712061

[ref44] Lupindo B. M. Maw A. Shabalala N. (2022). Late diagnosis of autism: exploring experiences of males diagnosed with autism in adulthood. Curr. Psychol. 42, 24181–24197. doi: 10.1007/s12144-022-03514-z, PMID: 35967496 PMC9361958

[ref45] Maciel Braga L. A. Mota F. B. (2021). Early cancer diagnosis using lab-on-a-chip devices: A bibliometric and network analysis. Collnet J. Scientometrics Inf. Manag. 15, 163–196. doi: 10.1080/09737766.2021.1949949

[ref46] Malik-Soni N. Shaker A. Luck H. Mullin A. E. Wiley R. E. Lewis M. E. S. . (2022). Tackling healthcare access barriers for individuals with autism from diagnosis to adulthood. Pediatr. Res. 91, 1028–1035. doi: 10.1038/s41390-021-01465-y, PMID: 33767375 PMC7993081

[ref47] Maljaars J. Gijbels E. Evers K. Spain D. Rumball F. Happé F. . (2023). Impact of the COVID-19 pandemic on daily life. Div. Exp. Autist. Adults 53, 319–331. doi: 10.1007/s10803-022-05437-x, PMID: 35076831 PMC8788399

[ref48] Mazurek M. O. (2014). Loneliness, friendship, and well-being in adults with autism spectrum disorders. Autism 18, 223–232. doi: 10.1177/1362361312474121, PMID: 24092838

[ref49] Menezes M. Harkins C. Robinson M. F. Pappagianopoulos J. Cross R. Vasa R. A. . (2022). Treatment of anxiety in autistic adults: a systematic review. Res. Autism Spectr. Disord. 99:102068. doi: 10.1016/j.rasd.2022.102068

[ref50] Moral-Muñoz J. A. Herrera-Viedma E. Santisteban-Espejo A. Cobo M. J. (2020). Software tools for conducting bibliometric analysis in science: an up-to-date review. El Profesional Inform. 29:03. doi: 10.3145/epi.2020.ene.03

[ref51] Morgan B. Nageye F. Masi G. Cortese S. (2020). Sleep in adults with autism Spectrum disorder: a systematic review and meta-analysis of subjective and objective studies. Sleep Med. 65, 113–120. doi: 10.1016/j.sleep.2019.07.019, PMID: 31739229

[ref52] Moseley R. L. Turner-Cobb J. M. Spahr C. M. Shields G. S. Slavich G. M. (2021). Lifetime and perceived stress, social support, loneliness, and health in autistic adults. Health Psychol. 40, 556–568. doi: 10.1037/hea0001108 34618502 PMC8513810

[ref53] Mota F. B. Braga L. A. M. Cabral B. P. Lopes R. M. Alves L. A. (2022). The scientific publication of the Memórias do Instituto Oswaldo Cruz (1909-2020): a history of contribution to the biomedical sciences. Mem. Inst. Oswaldo Cruz 117:e210376. doi: 10.1590/0074-02760210376, PMID: 35703661 PMC9196065

[ref54] Nepo K. Tincani M. Axelrod S. (2021). Teaching Mobile device-based leisure to adults with autism Spectrum disorder and intellectual disability. Focus Autism Other Dev. Disabl. 36, 83–94. doi: 10.1177/1088357620943500

[ref55] Nicolaidis C. Schnider G. Lee J. Raymaker D. M. Kapp S. K. Croen L. A. . (2021a). Development and psychometric testing of the AASPIRE adult autism healthcare provider self-efficacy scale. Autism 25, 767–773. doi: 10.1177/1362361320949734, PMID: 32859135 PMC8204689

[ref56] Nicolaidis C. Zhen K. Y. Lee J. Raymaker D. M. Kapp S. K. Croen L. A. . (2021b). Psychometric testing of a set of patient-reported instruments to assess healthcare interventions for autistic adults. Autism 25, 786–799. doi: 10.1177/1362361320967178, PMID: 33103457 PMC8068734

[ref57] Nocon A. S. Roestorf A. Menéndez L. M. G. (2022). Positive psychology in neurodiversity: an investigation of character strengths in autistic adults in the United Kingdom in a community setting. Res. Autism Spectr. Disord. 99:102071. doi: 10.1016/j.rasd.2022.102071

[ref58] Normansell-Mossa K. M. Top D. N. Russell N. Freeston M. Rodgers J. South M. (2021). Sensory sensitivity and intolerance of uncertainty influence anxiety in autistic adults. Front. Psychol. 12:731753. doi: 10.3389/fpsyg.2021.731753, PMID: 34867612 PMC8635111

[ref59] O’Nions E. Petersen I. Buckman J. E. J. Charlton R. Cooper C. Corbett A. . (2023). Autism in England: assessing underdiagnosis in a population-based cohort study of prospectively collected primary care data. Lancet Region. Health 29:100626. doi: 10.1016/j.lanepe.2023.100626, PMID: 37090088 PMC10114511

[ref60] Okoye C. Obialo-Ibeawuchi C. M. Obajeun O. A. Sarwar S. Tawfik C. Waleed M. S. . (2023). Early diagnosis of autism Spectrum disorder: a review and analysis of the risks and benefits. Cureus 15:e43226. doi: 10.7759/cureus.43226, PMID: 37692637 PMC10491411

[ref61] Oliveros J. C. Santiesteban I. Ulloa J. L. (2023). Can measures of social cognition predict autistic traits? Acta Psychol. 240:104056. doi: 10.1016/j.actpsy.2023.104056, PMID: 37865000

[ref62] Page M. J. McKenzie J. E. Bossuyt P. M. Boutron I. Hoffmann T. C. Mulrow C. D. . (2021). The PRISMA 2020 statement: an updated guideline for reporting systematic reviews. BMJ 372:71. doi: 10.1136/bmj.n71, PMID: 33782057 PMC8005924

[ref63] Qutub N. A. (2023). Parental attitudes toward the marriage of adult children with autism spectrum disorder and mental disability. Int. J. Adv. Appl. Sci. 10, 205–209. doi: 10.21833/ijaas.2023.03.025

[ref64] Rasero J. Jimenez-Marin A. Diez I. Toro R. Hasan M. T. Cortes J. M. (2023). The Neurogenetics of functional connectivity alterations in autism: insights from subtyping in 657 individuals. Biol. Psychiatry 94, 804–813. doi: 10.1016/j.biopsych.2023.04.014, PMID: 37088169

[ref65] Raul P. McNally K. Ward L. M. van Boxtel J. J. A. (2023). Does stochastic resonance improve performance for individuals with higher autism-spectrum quotient? Front. Neurosci. 17:1110714. doi: 10.3389/fnins.2023.1110714, PMID: 37123379 PMC10140507

[ref66] Roman-Urrestarazu A. Van Kessel R. Allison C. Matthews F. E. Brayne C. Baron-Cohen S. (2021). Association of Race/ethnicity and social disadvantage with autism prevalence in 7 million school children in England. JAMA Pediatr. 175:e210054. doi: 10.1001/jamapediatrics.2021.0054, PMID: 33779707 PMC8008434

[ref67] Rumball F. Happé F. Grey N. (2020). Experience of trauma and PTSD symptoms in autistic adults: risk of PTSD development following DSM-5 and non-DSM-5 traumatic life events. Autism Res. 13, 2122–2132. doi: 10.1002/aur.2306, PMID: 32319731

[ref68] Russell A. J. Murphy C. M. Wilson E. Gillan N. Brown C. Robertson D. M. . (2016). The mental health of individuals referred for assessment of autism spectrum disorder in adulthood: a clinic report. Autism 20, 623–627. doi: 10.1177/1362361315604271, PMID: 26471427

[ref69] Salari N. Rasoulpoor S. Rasoulpoor S. Shohaimi S. Jafarpour S. Abdoli N. . (2022). The global prevalence of autism spectrum disorder: a comprehensive systematic review and meta-analysis. Ital. J. Pediatr. 48:112. doi: 10.1186/s13052-022-01310-w 35804408 PMC9270782

[ref70] Scheeren A. M. Crane L. Heyworth M. Pellicano E. (2023). Impact of the COVID-19 pandemic on autistic adults: a scoping review. Curr. Dev. Disord. Rep. 10, 92–122. doi: 10.1007/s40474-023-00268-6, PMID: 36741810 PMC9887236

[ref71] Schwartzman J. M. Corbett B. A. (2022). Depression and employment outcomes in autistic adults: a systematic review. Rev. J. Autism Dev. Disord. 11, 157–171. doi: 10.1007/s40489-022-00331-9

[ref72] Solmi M. Song M. Yon D. K. Lee S. W. Fombonne E. Kim M. S. . (2022). Incidence, prevalence, and global burden of autism spectrum disorder from 1990 to 2019 across 204 countries. Mol. Psychiatry 27, 4172–4180. doi: 10.1038/s41380-022-01630-7, PMID: 35768640

[ref73] Song W. Nonnemacher S. L. Miller K. K. Anderson K. Shea L. L. (2022). Living arrangements and satisfaction of current arrangement among autistic adults reported by autistic individuals or their caregivers. J. Appl. Res. Intellect. Disabil. 35, 1174–1185. doi: 10.1111/jar.13003 35570334

[ref74] Speyer R. Chen Y. W. Kim J. H. Wilkes-Gillan S. Nordahl-Hansen A. J. Wu H. C. . (2022). Non-pharmacological interventions for adults with autism: a systematic review of randomised controlled trials. Rev. J. Autism Dev. Disord. 9, 249–279. doi: 10.1007/s40489-021-00250-1

[ref75] Talantseva O. I. Romanova R. S. Shurdova E. M. Dolgorukova T. A. Sologub P. S. Titova O. S. . (2023). The global prevalence of autism spectrum disorder: a three-level meta-analysis. Front. Psych. 14:1071181. doi: 10.3389/fpsyt.2023.1071181, PMID: 36846240 PMC9947250

[ref76] Taylor J. L. Adams R. E. Pezzimenti F. Zheng S. Bishop S. L. (2022). Job loss predicts worsening depressive symptoms for young adults with autism: a COVID−19 natural experiment. Autism Res. 15, 93–102. doi: 10.1002/aur.2621, PMID: 34626164 PMC8646555

[ref77] Thompson C. Brook M. Hick S. Miotti C. Toong R. McVeigh J. (2023). Physical activity, sedentary behaviour and their correlates in adults with autism Spectrum disorder: a systematic review. Rev. J. Autism Dev. Disord. 10, 546–562. doi: 10.1007/s40489-022-00305-x

[ref78] Underwood J. F. G. DelPozo-Banos M. Frizzati A. Rai D. John A. Hall J. (2023). Neurological and psychiatric disorders among autistic adults: a population healthcare record study. Psychol. Med. 53, 5663–5673. doi: 10.1017/S0033291722002884, PMID: 36189783 PMC10482712

[ref79] Wehman P. Brooke V. Brooke A. M. Ham W. Schall C. McDonough J. . (2016). Employment for adults with autism spectrum disorders: a retrospective review of a customized employment approach. Res. Dev. Disabil. 53-54, 61–72. doi: 10.1016/j.ridd.2016.01.015, PMID: 26855048

[ref80] WHO (2023). WHO key facts. Available at: https://www.who.int/news-room/fact-sheets/detail/autism-spectrum-disorders (Accessed December 3, 2023).

[ref81] World Health Organization (1992). The ICD-10 classification of mental and behavioural disorders: Clinical descriptions and diagnostic guidelines. Geneva: World Health Organization.

[ref82] World Health Organization (2013). Meeting report: autism spectrum disorders and other developmental disorders: from raising awareness to building capacity. Available at: https://iris.who.int/bitstream/handle/10665/103312/9789241506618_eng.pdf?sequence=1 (Accessed November 25, 2023).

[ref83] Zeidan J. Fombonne E. Scorah J. Ibrahim A. Durkin M. S. Saxena S. . (2022). Global prevalence of autism: a systematic review update. Autism Res. 15, 778–790. doi: 10.1002/aur.2696, PMID: 35238171 PMC9310578

