# Supplementary material for: Exploring the landscape of adult autism research in psychology: a bibliometric and network analysis
Source: Front Psychol. 2024 Sep 12;15:1427090. doi: 10.3389/fpsyg.2024.1427090 (PMC11424422; doi:10.3389/fpsyg.2024.1427090)
Supplement: Supplementary file 1 [file Table_1.DOCX]

Supplementary Material

# Network analysis’s centrality metrics Tables

**Supplementary Table 1.** Network of keywords

| **Keywords** | **DC** | **CC** | **BC** | **EC** | **WDC** |
| --- | --- | --- | --- | --- | --- |
| Emotion | 22 | 0.72 | 0.073796 | 0.822023 | 110 |
| Anxiety | 25 | 0.765957 | 0.07371 | 1 | 132 |
| Mental health | 22 | 0.72 | 0.060939 | 0.891994 | 118 |
| Depression | 23 | 0.734694 | 0.049362 | 0.956458 | 140 |
| Quality of life | 20 | 0.692308 | 0.042034 | 0.808962 | 74 |
| Intervention | 17 | 0.654545 | 0.03226 | 0.70661 | 58 |
| Empathy | 12 | 0.6 | 0.029056 | 0.382111 | 52 |
| Social cognition | 15 | 0.631579 | 0.028993 | 0.541927 | 78 |
| Intellectual disability | 18 | 0.666667 | 0.026161 | 0.786571 | 72 |
| Aging | 17 | 0.654545 | 0.025861 | 0.720466 | 82 |
| Qualitative research | 15 | 0.631579 | 0.024044 | 0.646292 | 54 |
| Employment | 16 | 0.631579 | 0.022211 | 0.672429 | 58 |
| Cognition | 13 | 0.610169 | 0.019697 | 0.490258 | 62 |
| Diagnosis | 15 | 0.62069 | 0.017818 | 0.63953 | 44 |
| Theory of mind | 12 | 0.590164 | 0.015484 | 0.399545 | 72 |
| Gender | 14 | 0.62069 | 0.014598 | 0.61957 | 40 |
| Social skills | 11 | 0.590164 | 0.011864 | 0.473114 | 42 |
| High-functioning autism | 11 | 0.580645 | 0.011751 | 0.48315 | 28 |
| COVID-19 | 14 | 0.62069 | 0.011135 | 0.642118 | 40 |
| Health services | 14 | 0.610169 | 0.010755 | 0.634214 | 46 |
| Memory | 9 | 0.5625 | 0.009945 | 0.371095 | 54 |
| Healthcare | 9 | 0.529412 | 0.009652 | 0.336143 | 20 |
| Comorbidity | 14 | 0.62069 | 0.00958 | 0.665469 | 78 |
| Self-report | 8 | 0.545455 | 0.009248 | 0.312756 | 16 |
| Alexithymia | 11 | 0.590164 | 0.008563 | 0.459294 | 34 |
| Executive function | 12 | 0.590164 | 0.007292 | 0.558536 | 54 |
| Social interaction | 8 | 0.545455 | 0.006817 | 0.25979 | 24 |
| Transition | 10 | 0.571429 | 0.005931 | 0.440235 | 34 |
| Well-being | 9 | 0.571429 | 0.005425 | 0.426371 | 30 |
| Virtual Reality | 7 | 0.537313 | 0.005098 | 0.269068 | 24 |
| Sleep | 10 | 0.5625 | 0.004621 | 0.464853 | 20 |
| Neurodiversity | 6 | 0.507042 | 0.004218 | 0.200671 | 12 |
| Stress | 10 | 0.571429 | 0.003908 | 0.485227 | 36 |
| Suicidality | 10 | 0.580645 | 0.00386 | 0.486684 | 30 |
| Eye tracking | 6 | 0.5 | 0.003577 | 0.195024 | 18 |
| Coping | 9 | 0.571429 | 0.003269 | 0.417459 | 26 |
| Sex differences | 8 | 0.521739 | 0.002227 | 0.369226 | 16 |

**Supplementary Table 2.** Network of Research Areas

|  | DC | WDC | CC | BC | EC |
| --- | --- | --- | --- | --- | --- |
| Psychology | 16 | 1138 | 1 | 0.7125 | 1 |
| Rehabilitation | 4 | 648 | 0.571429 | 0.002778 | 0.483688 |
| Education & Educational Research | 4 | 544 | 0.571429 | 0.008333 | 0.439307 |
| Psychiatry | 9 | 514 | 0.695652 | 0.101389 | 0.744785 |
| Behavioral Sciences | 3 | 274 | 0.551724 | 0.002778 | 0.340657 |
| Neurosciences & Neurology | 6 | 120 | 0.615385 | 0.036111 | 0.552505 |
| Linguistics | 2 | 24 | 0.533333 | 0 | 0.255203 |
| Pediatrics | 3 | 20 | 0.551724 | 0.002778 | 0.369252 |
| Ophthalmology | 2 | 20 | 0.533333 | 0 | 0.275268 |
| Pharmacology & Pharmacy | 2 | 16 | 0.533333 | 0 | 0.308624 |
| Physiology | 2 | 16 | 0.533333 | 0 | 0.275268 |
| Criminology & Penology | 3 | 8 | 0.551724 | 0 | 0.374834 |
| Government & Law | 3 | 6 | 0.551724 | 0 | 0.374834 |
| Geriatrics & Gerontology | 2 | 4 | 0.533333 | 0 | 0.308624 |
| Music | 1 | 4 | 0.516129 | 0 | 0.177703 |
| Family Studies | 1 | 2 | 0.516129 | 0 | 0.177703 |
| Substance Abuse | 1 | 2 | 0.516129 | 0 | 0.177703 |

**Supplementary Table 3.** Network of Countries

| **Countries** | **DC** | **WDC** | **CC** | **BC** | **EC** |
| --- | --- | --- | --- | --- | --- |
| USA | 29 | 864 | 0.691994 | 0.728814 | 0.247209 |
| UK | 35 | 780 | 1 | 0.811321 | 0.323836 |
| Australia | 12 | 312 | 0.322337 | 0.565789 | 0.015133 |
| Germany | 23 | 234 | 0.874508 | 0.661538 | 0.040695 |
| Netherlands | 10 | 158 | 0.288613 | 0.551282 | 0.010253 |
| Sweden | 18 | 156 | 0.803391 | 0.614286 | 0.00831 |
| France | 21 | 154 | 0.84049 | 0.641791 | 0.036247 |
| Canada | 10 | 148 | 0.328642 | 0.551282 | 0.008673 |
| Spain | 18 | 118 | 0.803391 | 0.614286 | 0.00831 |
| Ireland | 19 | 92 | 0.810527 | 0.623188 | 0.021616 |
| Italy | 18 | 90 | 0.797927 | 0.614286 | 0.008588 |
| Denmark | 17 | 90 | 0.74737 | 0.551282 | 0.00407 |
| Finland | 18 | 76 | 0.774613 | 0.558442 | 0.005412 |
| Portugal | 16 | 76 | 0.735873 | 0.544304 | 0 |
| Austria | 18 | 72 | 0.797927 | 0.614286 | 0.008588 |
| Bulgaria | 16 | 60 | 0.735873 | 0.544304 | 0 |
| Iceland | 16 | 60 | 0.735873 | 0.544304 | 0 |
| Poland | 16 | 60 | 0.735873 | 0.544304 | 0 |
| Romania | 16 | 60 | 0.735873 | 0.544304 | 0 |
| Belgium | 8 | 60 | 0.302635 | 0.5375 | 8.33E-04 |
| Taiwan | 7 | 42 | 0.126947 | 0.472527 | 0.002035 |
| Argentina | 14 | 38 | 0.231688 | 0.581081 | 0.033333 |
| Switzerland | 5 | 32 | 0.163247 | 0.483146 | 0 |
| Japan | 4 | 32 | 0.079018 | 0.457447 | 0 |
| China | 4 | 30 | 0.103195 | 0.462366 | 0 |
| Singapore | 6 | 26 | 0.15987 | 0.52439 | 0 |
| Israel | 5 | 26 | 0.0929 | 0.438776 | 6.94E-04 |
| Chile | 9 | 24 | 0.170152 | 0.544304 | 0.017137 |
| New Zealand | 7 | 22 | 0.24575 | 0.530864 | 3.70E-04 |
| Brazil | 7 | 20 | 0.099442 | 0.447917 | 0 |
| India | 8 | 18 | 0.162569 | 0.530864 | 0.009019 |
| Mexico | 5 | 14 | 0.149854 | 0.467391 | 0 |
| Bangladesh | 7 | 12 | 0.098632 | 0.443299 | 0 |
| Dominican Rep | 7 | 12 | 0.099442 | 0.447917 | 0 |
| Kenya | 7 | 12 | 0.098632 | 0.443299 | 0 |
| Pakistan | 7 | 12 | 0.098632 | 0.443299 | 0 |
| Uruguay | 7 | 12 | 0.099442 | 0.447917 | 0 |
| Russia | 5 | 12 | 0.175593 | 0.467391 | 0 |
| Cyprus | 5 | 8 | 0.099605 | 0.462366 | 0 |
| Turkey | 3 | 8 | 0.069026 | 0.452632 | 0 |
| Hungary | 4 | 6 | 0.118184 | 0.505882 | 0 |
| Greece | 3 | 6 | 0.069026 | 0.452632 | 0 |
| Jordan | 3 | 4 | 0.050001 | 0.425743 | 0 |
| Malaysia | 3 | 4 | 0.069026 | 0.452632 | 0 |
| Costa Rica | 2 | 2 | 8.43E-04 | 0 | 0 |
| Indonesia | 2 | 2 | 8.43E-04 | 0 | 0 |
| Luxembourg | 2 | 2 | 8.43E-04 | 0 | 0 |
| South Africa | 2 | 2 | 8.43E-04 | 0 | 0 |

**Supplementary Table 4.** Network of Research Organizations

| **Organizations** | **DC** | **WDC** | **EC** | **CC** | **BC** |
| --- | --- | --- | --- | --- | --- |
| Kings Coll London, England | 14 | 110 | 1 | 0.586207 | 0.146666 |
| Univ Calif, USA | 10 | 42 | 0.435558 | 0.557377 | 0.223461 |
| Univ Coll London, England | 11 | 66 | 0.779845 | 0.523077 | 0.067427 |
| Univ Cambridge, England | 11 | 62 | 0.674963 | 0.586207 | 0.210264 |
| Univ New South Wales, Australia | 12 | 62 | 0.9797 | 0.53125 | 0.079719 |
| City Univ London, England | 6 | 22 | 0.449131 | 0.447368 | 0.012209 |
| Univ Amsterdam, Netherlands | 8 | 30 | 0.532195 | 0.5 | 0.055172 |
| Newcastle Univ, England | 11 | 34 | 0.728753 | 0.53125 | 0.076948 |
| Univ Bath, England | 4 | 28 | 0.345315 | 0.409639 | 0.001641 |
| South London & Maudsley, England | 4 | 44 | 0.316059 | 0.441558 | 0.007595 |
| Karolinska Inst, Sweden | 4 | 28 | 0.349263 | 0.409639 | 0.003726 |
| Univ North Carolina, USA | 6 | 14 | 0.409504 | 0.478873 | 0.022805 |
| Vrije Univ Amsterdam, Netherlands | 8 | 32 | 0.634713 | 0.492754 | 0.071971 |
| Macquarie Univ, Australia | 9 | 32 | 0.773372 | 0.472222 | 0.024283 |
| Univ Western Australia, Australia | 9 | 38 | 0.750682 | 0.472222 | 0.024951 |
| Univ Wisconsin, USA | 5 | 26 | 0.168093 | 0.441558 | 0.102673 |
| Vanderbilt Univ, USA | 3 | 26 | 0.094067 | 0.377778 | 0.00112 |
| Univ Texas Dallas, USA | 3 | 12 | 0.136019 | 0.414634 | 0.013265 |
| Stanford Univ, USA | 6 | 34 | 0.471239 | 0.478873 | 0.04552 |
| Univ Missouri, USA | 3 | 8 | 0.078875 | 0.373626 | 0.011657 |
| Portland State Univ, USA | 2 | 6 | 0.064305 | 0.377778 | 0.018543 |
| Univ Nottingham, England | 4 | 22 | 0.295475 | 0.43038 | 0.002131 |
| Univ Toronto, Canada | 2 | 18 | 0.178995 | 0.404762 | 0 |
| Univ Pittsburgh, USA | 1 | 6 | 0.024136 | 0.309091 | 0 |
| Univ Massachusetts Lowell, USA | 2 | 4 | 0.036797 | 0.314815 | 0.00112 |
| Univ Edinburgh, Scotland | 3 | 8 | 0.21093 | 0.404762 | 0 |
| Free Univ Berlin, Germany | 3 | 22 | 0.119382 | 0.409639 | 0.027939 |
| Drexel Univ, USA | 2 | 8 | 0.037381 | 0.330097 | 0.005882 |
| George Washington Univ, USA | 6 | 18 | 0.194551 | 0.425 | 0.07996 |
| La Trobe Univ, Australia | 7 | 50 | 0.562048 | 0.43038 | 0.00898 |
| Cooperat Res Ctr Living Autism, Australia | 8 | 54 | 0.600226 | 0.404762 | 0.00648 |
| Curtin Univ, Australia | 7 | 40 | 0.548873 | 0.377778 | 0.002577 |
| Univ Queensland, Australia | 7 | 40 | 0.562521 | 0.4 | 0.002558 |
| Radboud Univ Nijmegen, Netherlands | 3 | 12 | 0.198885 | 0.369565 | 4.20E-04 |
| Humboldt Univ, Germany | 2 | 18 | 0.044251 | 0.32381 | 0 |
| Ohio State Univ, USA | 0 | 0 | 0 | 0 | 0 |

# The search strategy of subsection 2.1 Limitations of the study

For # Web of Science Search Strategy (v0.1)

# Database: Web of Science Core Collection

# Entitlements:

- WOS.SCI: 1945 to 2023

- WOS.AHCI: 1975 to 2023

- WOS.ESCI: 2018 to 2023

- WOS.ISTP: 1991 to 2023

- WOS.SSCI: 1956 to 2023

- WOS.ISSHP: 1991 to 2023

# Searches:

1: TI=((“Autism*” or “Autistic*” or “Asperger*” or "ASD") near/3 (“Adult*”)) not (TI=(“Newborn*” or “Neonate*” or “Infant*” or “Child*” or “Adolescen*” or “Teen*” or “Youth*”) or TI=("*Broad* Autis* Phenotype*" or "*Broad* Autis* Spectrum*" or "autis* trait*" or "Autis* Spectrum* Trait*" or "Autis* Phenotype* trait*")) and Article (Document Types) and 2013 or 2014 or 2015 or 2016 or 2017 or 2018 or 2019 or 2020 or 2021 or 2022 (Publication Years) and Early Access (Exclude – Document Types)

Date Run: Thu Nov 23 2023; Results: 1547

2: TI=((“Autism*” or “Autistic*” or “Asperger*” or "ASD") near/3 (“Adult*”)) not (TI=(“Newborn*” or “Neonate*” or “Infant*” or “Child*” or “Adolescen*” or “Teen*” or “Youth*”) or TI=("*Broad* Autis* Phenotype*" or "*Broad* Autis* Spectrum*" or "autis* trait*" or "Autis* Spectrum* Trait*" or "Autis* Phenotype* trait*")) and Article (Document Types) and 2013 or 2014 or 2015 or 2016 or 2017 or 2018 or 2019 or 2020 or 2021 or 2022 (Publication Years) and Early Access (Exclude – Document Types) and Psychology (Research Areas)

Date Run: Thu Nov 23 2023; Results: 936

3: #1 NOT #2

Date Run: Thu Nov 23 2023; Results: 611
